# Altered Hub Configurations within Default Mode Network following Acupuncture at ST36: A Multimodal Investigation Combining fMRI and MEG

**DOI:** 10.1371/journal.pone.0064509

**Published:** 2013-05-17

**Authors:** Youbo You, Lijun Bai, Ruwei Dai, Hao Cheng, Zhenyu Liu, Wenjuan Wei, Jie Tian

**Affiliations:** 1 Key Laboratory of Molecular Imaging and Functional Imaging, Institute of Automation, Chinese Academy of Sciences, Beijing, China; 2 Life Science Research Center, School of Electronic Engineering, Xidian University, Xi'an, Shaanxi, China; 3 Department of Anesthesiology, Beijing Ditan Hospital affiliated to Capital Medical University, Beijing, China; Institute of Psychology, Chinese Academy of Sciences, China

## Abstract

Acupuncture, an externally somatosensory stimulation in the Traditional Chinese Medicine, has been proposed about its modulations on the brain's default mode network (DMN). However, it is still unknown on how the internal brain resting networks are modulated and what inferences can be made about the physiological processes underlying these changes. Combining high spatial resolution of functional magnetic resonance imaging (fMRI) with high temporal resolution of magnetoencephalography (MEG), in the current multimodal study, we sought to explore spatiotemporally whether or not band-specific DMN hub configurations would be induced by verum acupuncture, compared with sham control. Spatial independent component analysis was applied to fMRI data, followed by the discrete regional sources seeded into MEG data. Partial correlation analysis was further adopted to estimate the intrinsic functional connectivity and network hub configurations. One of the most striking findings is that the posterior cingulate cortex is not only validated as a robust DMN hub, but served as a hub only within the delta and gamma bands following the verum acupuncture, compared with its consistently being a DMN hub in sham control group. Our preliminary results may provide a new perspective to lend support for the specificity of neural mechanism underlying acupuncture.

## Introduction

Acupuncture, an ancient healing modality in the Traditional Chinese Medicine, has the therapeutic effects in the treatment of a range of diverse disorders [1]–[2]. However, it has not gained a proper position so far in the modern biomedical disciplines. This is partly due to the fact that physiological mechanisms underlying acupuncture effects still remain elusive. Among all research interests, one of the most appealing is focused on the unresolved but fundamental issue in which the central representations of peripheral acupuncture stimulation with regard to its functional specificity. In other words, whether exerting acupuncture at certain acupoints can produce functionally specific effects in the brain compared to a sham or placebo control procedure. Although accumulating evidence emerges [3]–[7], the debate over such acupoint specificity continues [8]–[10]. In order to promote better acceptance of acupuncture as a viable clinical treatment, it is thus vital and necessary to explore the biological mechanisms of functional specificity underlying acupuncture.

It is remarkable to find that a large proportion of neuroimaging acupuncture researches have been carried out with the utilization of functional magnetic resonance imaging (fMRI). Indeed, its high spatial resolution (on the order of millimeters scale) has prominently paved the way to identify the induced activity of brain regions in the spatial dimension reliably [5]. However, the Blood Oxygen Level Dependent signal detected by fMRI only reflects the neuronal activity indirectly [11]–[12]. Due to the latency of the hemodynamic response, it is more or less handicapped in the temporal dimension, only examining correlations in the relatively slow neuronal oscillations [13]. Analysis conducted only within this low frequency domain in previous acupuncture researches thereby may be limited to unveil completely the underlying mechanism since most electrophysiological aspects of neural activities took place at a much faster time scale [14]. For instance, working memory is associated with neuron interactions in the theta band, while gamma synchronization is related to perception and consciousness [15]–[16]. Electrophysiological imaging technology such as magnetoencephalography (MEG), on the contrary, has the ability to bypass the hemodynamic response and measure the magnetic fields induced by synchronized current flows in neuronal assemblies [17]. It can provide a unique window into the neurophysiological processing on the milliseconds time scale [18] so as to capture the detailed temporal profile of neural responses induced by acupuncture. However, its spatial resolving power does not match that of fMRI [19]. Since fMRI offers excellent spatial resolution with poor temporal resolution, while MEG provides excellent temporal resolution but poor spatial resolution, one feasible idea occurred that more exhaustive understanding of neural processes would be achieved by the combined employment of fMRI for localization and MEG for timing [20]–[22].

During the last decade, it has been gradually recognized that the brain is a complex network of dynamic systems with abundant functional interactions between local and remote regions [23]–[26]. One of the fast growing interests is related to the default mode network (DMN), which is identified as the distributed brain regions activated during rest but deactivated when specific goal-directed behavior is needed [27]–[28]. Accumulating evidence has typically demonstrated that several DMN regions play pivotal roles in connecting other regions and serve as network hubs with significant higher functional connectivity density [23], [29]. In particular, recent investigations disclosed that the posterior cingulated cortex (PCC) constitutes one of the strongest DMN hubs in healthy subjects, with the highest degree of connectivity with other regions [29]–[32]. While substantial information has been gained about the prominent role of the hubs, the issues require more investigations on how these hubs interact with the rest of DMN regions and, more importantly, their alterations induced by acupuncture. The function of DMN has been considered to putatively engaged in self referential mental activity [33], stimulus-independent thoughts [34], and monitoring the environment among others [35] to maintain the body's homeostasis. In practice, the well-identified physical effects of acupuncture needling and its purported clinical efficacy also suggest that acupuncture acts in maintaining a homeostatic balance of the internal state within and across multiple brain networks [36]. Since the whole-brain hubs could be rearranged specifically following verum acupuncture compared with sham stimulation [37], furthermore, it has been increasingly elucidated that acupuncture could modulate spontaneous neural activities in wide resting brain networks, particularly within the DMN and its anticorrelated networks [5], [38]–[40], therefore, further understanding of how such external intervention interacts with internal regulatory processes by regulating the DMN hubs may enlighten us to gain an appreciation of the physiological function and integrated mechanisms involved in acupuncture [5]. In recent electrophysiological studies, it is newly elucidated that functional connectivity between whole-brain regions would be modulated by acupuncture within specific frequency bands [41].

The current study attempted to spatiotemporally explore the specific DMN hub configurations following the verum acupuncture (Stomach Meridian 36, ST36), using sham acupuncture (non-meridian point, NAP) as a control. Moreover, since previous investigations demonstrated the modulation on band-specific functional connectivity among the whole-brain network, it is proposed here that whether such regulations would specifically exist within conventional frequency bands, that is, delta (0.5–4 Hz), theta (4–8 Hz), alpha (8–13 Hz), beta (13–30 Hz) and gamma bands (30–48 Hz). To test this hypothesis, we attempted to employ fMRI for spatially identifying the DMN component and MEG for temporally evaluating the hub configurations so that more comprehensive knowledge would be dug out on the specific physiological mechanism underlying acupuncture.

## Materials and Methods

### Subjects

In order to reduce inter-subject difference, 28 right-handed healthy college students (14 males, 14 females, aged 24.5±1.8 years) from a homogeneous group were enrolled. All of them were acupuncture naïve. Participants were screened to exclude individuals with a history of major medical illness, head trauma, neuropsychiatric disorders or used any prescription medications within the last month. All subjects gave written, informed consent after the experimental procedures being fully explained. The Tiantan Hospital Subcommittee on Human Studies approved the methods and procedures, all of which was in accordance with the Declaration of Helsinki.

### Experimental paradigm

**Figure 1:**
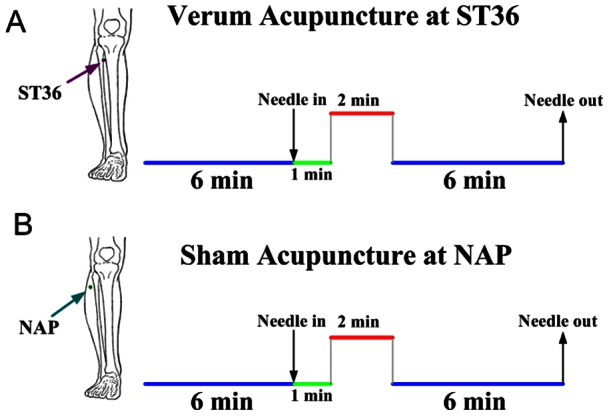
Experimental paradigm. Panel A indicated that acupuncture stimulation was performed at acupoint ST36 on the right leg (Zusanli, arrow pointing to dark pink dot). Panel B indicated that needling was performed at a nearby nonacupoint on the right leg (NAP, arrow pointing to dark cyan dot). The red line refers to needle administration, and the green line represents no acupuncture manipulation with needle inserted, the blue line indicates a 6-minute resting state or post-acupuncture resting state.

Twenty-eight participants were evenly divided into two groups, one for ST36 and the other for NAP, being matched by age and gender. All participants underwent firstly resting state functional MRI scanning for 6 min, followed by MEG data collection, during which the manual acupuncture was exerted at either ST36 or NAP. The whole MEG data collection lasts for 15 min, with the first 6 min for resting-state scanning and the other 9 min for acupuncture intervention. Both verum and sham manual acupuncture employed the single-block design paradigm, incorporating 2 min needle manipulation, preceded by 1 min rest epoch and followed by 6 min rest (without acupuncture manipulation) scanning. The experimental paradigm of MEG scanning can be found in Fig. 1. All participants were asked to remain relaxed without engaging in any mental tasks. To facilitate blinding, they were also instructed to keep their eyes closed to prevent from actually observing the procedures.

Verum acupuncture was performed at ST36 on the right leg (Zusanli, located four finger breadths below the lower margin of the patella and one finger breadth laterally from the anterior crest of the tibia) [42]–[43]. Verum acupuncture was delivered using a sterile disposable 38 gauge stainless steel acupuncture needle, 0.2 mm in diameter and 40 mm in length, which was inserted perpendicularly into the skin surface at a depth of 1.5–2.5 cm. Sham acupuncture was initially devised by an experienced and licensed acupuncturist (with 6 years of experience and had been trained to perform in the fMRI settings), with needling at non-meridian point (2–3 cm apart from ST36), with needle depth, stimulation intensity and manipulation procedure all identical to those used in verum acupuncture. A balanced “tonifying and reducing” technique was utilized as twirling the needle clockwise and counter-clockwise equally [44]. Stimulation consisted of rotating the needle clockwise and counterclockwise for 1 min at a rate of 60 times per min. The whole procedure was performed by the same acupuncturist on all participants.

According to Traditional Chinese Medicine, the sensation induced by twirling needles at the acupoints is asserted as “*De-qi*”, which is essential to the efficacy of acupuncture [45]. As a concurrent psychophysical analysis, the MGH Acupuncture Sensation Scale (MASS) was utilized in the present study to quantify the subjective *De*-*qi* sensations [38], [46]. The sensation rates ranged from 0 to 10 (0 =  no sensation, 1–3 =  mild, 4–6 =  moderate, 7–8 =  strong, 9 =  severe and 10 =  unbearable sensation). Spreading of any sensation was noted in a binary fashion and coded as follows: 1-spreading reported; 0-spreading not reported.

### fMRI and MEG data acquisition

All fMRI data were obtained with a 3.0 T MRI system (Allegra; Siemens, Erlangen, Germany). A custom-built head holder and foam padding were used to restricted head movements. The images were parallel to the AC-PC line and covered the whole brain. Thirty-two axial slices were obtained using a T2*-weighted single-shot, gradient-recalled echo planar imaging sequence (FOV  = 240 mm×240 mm, matrix  = 64×64, thickness  = 5 mm, TR  = 2000 ms, TE  = 30 ms, flip angle  = 75°). The total functional scanning was lasted for 6 minutes during the resting state. After the functional run, high-resolution structural information on each subject was also acquired using 3D MRI sequences with a voxel size of 1 mm^3^ for anatomical localization (TR  = 2700 ms, TE  = 2.19 ms, matrix  = 384×512, FOV  = 256 mm×256 mm, flip angel  = 7°, thickness  = 1 mm).

The MEG data were recorded when participants comfortably seated inside an electromagnetically shielded room. The cortical responses were recorded with the whole head MEG system (CTF Systems Inc., Port Coquitlam, BC, Canada) consisting of 151 hardware first-order magnetic gradiometers. Two of the original 151 channels were not available due to technical problems during recording for all participants. The whole MEG scanning lasted 15 minutes, incorporating a 6 min resting state recording and followed by above-mentioned 9-min acupuncture procedure (Fig. 1). The head position was monitored during the measurement using head position indicator coils. MEG data were recorded at the sample rate of 600 Hz. The same acquisition settings were used for an empty-room recording without a subject in the MEG room to estimate the background noise [47]. During the recording, participants were instructed to close their eyes to reduce artifact signals due to eye movements, but remain awake as much as possible. The investigator and MEG technician checked the signal on-line and observed the participants using a video monitor. The head position relative to the MEG sensors was measured before and after each recording session by leading small alternating currents through three head position coils attached to the left and right pre-auricular points and the nasion on the subject's head. For all analyzed data sets, head displacements within a recording session were below 5 mm.

### fMRI data analysis

The first five volumes of fMRI data were discarded to eliminate non-equilibrium effects of magnetization [48]. All images were subsequently pre-processed using the statistical parametric mapping (SPM5, http://www.fil.ion.ucl.ac.uk/spm/) [49]. Firstly, the image data underwent realignments for head motions using the least-squares minimization. None of subjects had head movements exceeding 1 mm on any axis or head rotation greater than one degree. A mean image created from the realigned volumes was coregistered with the subject's individual structural T1-weighted volume image. Secondly, the standard Montreal Neurological Institute (MNI) template provided by SPM5 was used in the spatial normalization. Then these data were resampled at 2 mm×2 mm×2 mm and filtered utilizing a finite-impulse response band-pass filter (0.01∼0.08 Hz) in order to remove the effect of low-frequency drift and high-frequency noise [50]–[51]. Subsequently, the functional images were smoothed by a Gaussian kernel with a full width at half maximum of 6 mm (FWHM  = 6 mm).

**Figure 2:**
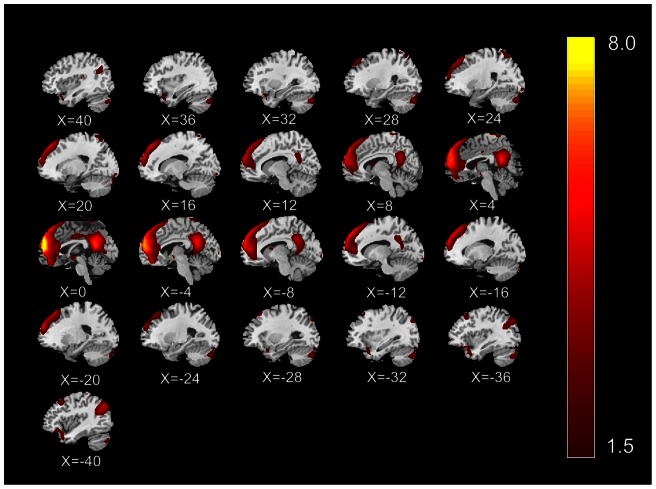
Spatial DMN components of group results extracted from fMRI data. Images are Z statistics overlaid on the average high-resolution scan transformed into standard (MNI 152) space. Red to yellow are Z values, ranging from 1.5 to 8.0.

A data-driven method named independent component analysis (ICA), which is able to extract multiple functional connectivity networks [26], [52], was performed on the preprocessed data of all subjects using the Group ICA of fMRI Toolbox (GIFT) [53]–[54]. To reduce the computational load of simply entering all subjects' data into an ICA analysis, two reduction steps were conducted, one on data from each subject and the other on an aggregate data set. In the first round of principal component analysis (PCA), the data for individual subject were dimension-reduced to 52 in the GIFT toolbox. After concatenation across subjects, the dimension was again reduced via the second round of PCA to 33 components estimated by the Minimum Description Length (MDL) criteria, followed by an independent component estimation using Infomax algorithm [54]–[55]. The mean ICs of all the subjects, the corresponding mean time courses and ICs for each subject were obtained from group ICA separation and back-reconstruction [53]. The maps of these ICs across all subjects were generated for a random effect analysis using a one-sample t-test. Thresholds were set at P<0.05 (correction with the FDR criterion). The intensity values in each spatial map were converted to Z scores to indicate the voxels that contributed most strongly to a particular IC. Voxels with absolute Z-values greater than 1.5 are considered active voxels of the IC [56]. The independent component was selected, which best matched the default mode network as previously reported [26], [28]. Every template region was spherical with a radius of 5 mm (varying sphere size had no effect on component identification), and the average value of voxels within the template minus that of voxels outside the template was calculated for each component. Finally, the component with the greatest difference was determined to be the best-fit component and was designated as the DMN. The peak voxels of 8 core regions of interest (ROIs) were subsequently identified within the DMN component. These ROIs mainly include the bilateral medial frontal gyrus (MFG), superior frontal gyrus (SFG), anterior cingulate cortex (ACC), posterior cingulate cortex (PCC), superior temporal gyrus (STG), angular gyrus (AG), inferior parietal lobule (IPL) and middle temporal gyrus (MTG) [50], [57]–[59]. See Fig. 2. Talairach coordinates of peak foci for each ROI are listed in the Table 1.

**Table 1:** Talairach coordinates of the regional sources used to model the MEG data (P<0.05, FDR corrected).

Regions of Interest	Hem	Talairach			Zvalue	Vvoxels
		x	y	z		
MFG	**L**	−2	57	21	5.54	422
	**R**	2	57	8	4.94	341
SFG	**L**	−2	57	23	5.57	495
	**R**	4	58	25	3.19	742
ACC	**L**	−2	54	−1	3.71	77
	**R**	4	47	9	2.75	57
PCC	**L**	−2	−49	25	3.14	65
	**R**	2	−50	19	3.36	82
STG	**L**	−38	17	−19	2.21	56
	**R**	38	17	−19	2.27	33
AG	**L**	−49	−63	31	2.43	19
	**R**	51	−63	31	2.00	16
IPL	**L**	−46	−50	52	2.40	206
	**R**	50	−42	56	1.52	15
MTG	**L**	−49	−63	29	2.41	39
	**R**	53	−59	25	2.10	22

Abbreviations: Hem, hemisphere; L, left; R, right; MFG, medial frontal gyrus; SFG, superior frontal gyrus; ACC, anterior cingulate cortex; PCC, posterior cingulate cortex/precuneus; STG, superior temporal gyrus; AG, angular gyrus; IPL, inferior parietal lobule; MTG, middle temporal gyrus.

### MEG data analysis

A third-order gradient noise reduction (computed with CTF software) was applied to the MEG signals on line. The raw data were then digitally filtered off-line with a band-pass of 0.5–48 Hz, followed by down-sampled at a rate of 300 Hz. Subsequently, the MEG data were band-passed into bands of interest: delta (0.5–4 Hz), theta (4–8 Hz), alpha (8–13 Hz), beta (13–30 Hz) and gamma band (30–48 Hz) [60]–[62]. To specifically highlight the temporal dynamics within and across different regions of the default mode network, discrete regional dipole source analysis [63] was applied in each frequency band to create a spatial filter to project into source space using the Brain Electrical Source Analysis (BESA, MEGIS Software GmbH, Germany) software package which implements a least squares algorithm to solve the overdetermined problem and estimate the activity contributed by each source to the scalp-recorded data [64]–[66]. This methodology overcomes some of the limitations associated with conducting analysis only in the sensor space and allows the spatiotemporal modeling of multiple simultaneous sources over defined intervals [67]. In our study, to represent brain activity within DMN preceding or following acupuncture, relevant fixed regional sources were seeded into a 3-layer spherical head model and source activity was estimated from each subject's continuous scalp data for further analysis [64], [68]. Positioning of regional sources drew on the spatial localization of peak voxels in the DMN component obtained in fMRI. Talairach coordinates of these regional sources and proximate cortical structures are listed in Table 1.

### Functional connectivity analysis and hub definition

Partial correlation analysis has been proven effective as a measure of the functional connectivity between a given pair of regions by attenuating the contribution of other sources of covariance [69]–[71]. Besides, partial correlations can be used to build undirected graphs, in which connections (edges) between nodes (vertices) depict their conditional dependence [69], [72]. Given a set of *N* random variables, the partial correlation matrix is a symmetric matrix in which each off-diagonal element is the correlation coefficient between a pair of variables after partialling out the contributions to the pairwise correlation of all other variables included in the dataset [71], [73]–[75]. In our case, it was thus utilized within each frequency band to estimate the correlation coefficient between each pair of regions within DMN, factoring out the contribution to the pairwise correlations of the other 14 brain regions.

To estimate the partial correlation matrix within each frequency band for every participant, covariance matrix *S* was generated, using the 360s-long MEG data matrix *Y* on a subject-by-subject basis. Each component of *S* contains the sample covariance value between two brain regions (*j*, *k*) was 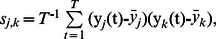 where 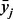 denotes the average over time of the observations in a given region, *T* denotes the number of time points. Afterwards, the off-diagonal elements of the inverted matrix 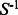 was rescaled to obtain the partial correlation matrix *R*: 
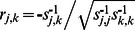
 [71]. A Fisher's *r*-to-*Z* transformation was then applied on the partial correlation matrices in order to improve the normality of the partial correlation coefficients [76]. To test the null hypothesis that the partial correlation was zero between any pair of regions within DMN, we conducted multiple one-sample *t*-tests for the individually estimated partial correlations for each condition. False discovery rate (FDR) procedure was applied to restrict the expected proportion of type I errors to *q*<0.05 [77]. Subsequently, the partial correlation matrices obtained individually were averaged to obtain group mean inter-regional functional connectivity. Due to the lack of consensus as to the best method for defining network hubs, a pragmatic method was applied in which nodes were identified as network hubs if their degree values were at least one standard deviation greater than the average degree of the network [37], [78]. Finally, significant alterations of functional connectivity induced by acupuncture for either ST36 or NAP group was evaluated for each band by means of a paired *t*-test with thresholded at *P*<0.05 in SPSS 17.0 software package for Windows.

## Results

### Psychophysical responses

**Figure 3:**
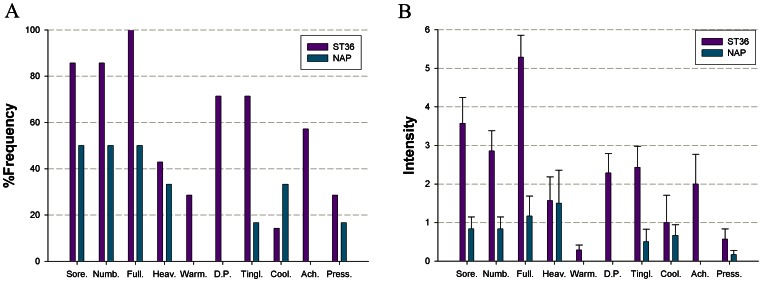
Averaged psychophysical response. A. The percentage of subjects that reported the given sensations. The frequency of aching was found greater following acupuncture at ST36. B. The intensity of sensations measured by average score (with standard error bars) on a scale from 0 denoting no sensation to 10 denoting an unbearable sensation. Sore, soreness; Numb, numbness; Full, fullness; Heav, heaviness; Warm, warmth; DP, dull pain; Tinl, tingling; Cool, coolness; Ach, aching; Press, pressure.

The prevalence of subjective “*De-qi*” sensations was expressed as the percentage of individuals in the group who reported the given sensations (Fig. 3A). The intensity was expressed as the average score ± standard error (Fig. 3B). The occurrence frequency of all sensations except coolness was found to be greater for acupuncture at ST36 than NAP. The overall stimulus intensities (mean ± SE) were greater for ST36, exhibiting a stronger *De-qi* sensation in verum acupuncture (*P*<0.05, two sample *t*-test).

### Band-specific DMN hub configurations

In the present study, the pairwise functional connectivity analysis between the 8 core brain regions within the default mode network were evaluated for each of the 5 conventional frequency bands (delta, theta, alpha, beta and gamma) preceding and following acupuncture, with the aim of exploring the band-specific alterations of DMN hub configurations induced by verum acupuncture at ST36, using a nearby non-acupoint (NAP, sham acupuncture) as a control. The summary of the DMN hub configurations within each frequency band was listed in the Table 2.

**Table 2:** The DMN hub configurations during the resting state (REST), following sham acupuncture (NAP) and verum acupuncture (ST36) within the 5 frequency bands respectively.

Frequency band	Conditions		
	REST	NAP	ST36
Delta	PCC, IPL	PCC, MTG	PCC, SFG
Theta	PCC	PCC, STG	MFG, STG, SFG
Alpha	PCC, ACC,STG, SFG	PCC, ACC,STG, SFG, MFG	STG, SFG
Beta	PCC, ACC, SFG	PCC, ACC, SFG	ACC, SFG
Gamma	PCC	PCC, ACC	PCC, STG

Abbreviations as in Table 1.

**Figure 4:**
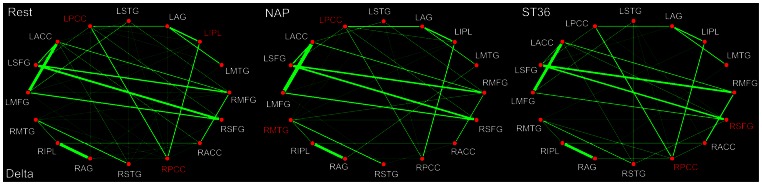
DMN hub configurations preceding acupuncture and following acupuncture at ST36 or NAP within the delta band (P<0.05, FDR corrected). The DMN hubs are indicated in red which are denoted as at least one standard deviation greater than the average degree of the network. The strength of the functional connectivity was expressed by the thickness of the green line. L, left; R, right; MFG, medial frontal gyrus; SFG, superior frontal gyrus; ACC, anterior cingulate cortex; PCC, posterior cingulate cortex/precuneus; STG, superior temporal gyrus; AG, angular gyrus; IPL, inferior parietal lobule; MTG, middle temporal gyrus.

For the delta band (0.5–4 Hz), the PCC and IPL were identified as DMN hubs during the resting state (Fig. 4). Following acupuncture, either at ST36 or at NAP, it is of interest to find that little was modulated on the key role played by PCC in the DMN hub configurations. By contrast, the IPL no longer acted as a network hub within the DMN in either group during the post-stimuli acupuncture resting state. In addition, specific acupuncture-induced alterations on hub configuration were also exhibited. To be specific, the right MTG was identified as a hub following sham acupuncture, while the right SFG was spotted following verum acupuncture at ST36. Taken into account the acupuncture-induced alterations of functional connectivity, it can be found that the functional interactions were suspended between STG and IPL, ACC and AG following either sham or verum acupuncture. In the NAP group, the connectivity between ACC and PCC as well as SFG and STG was found to be interrupted. On the contrary, the relation between MTG and MFG together with IPL and MFG was disconnected following acupuncture at ST36. Meanwhile, there emerged the functional connectivity between the SFG and AG as well as increased interaction between PCC and STG in the ST36 group (Table 3).

**Table 3:** The functional connectivity between the DMN key nodes and their alterations induced by acupuncture within the delta band (0.5–4 Hz).

Functional Connectivity	Delta					
	Rest	NAP	*P*-value	ST36	*P*-value	
MFG-SFG	0.3643	0.4011	0.358	0.4299	0.723	
SFG-ACC	0.2332	0.3016	0.231	0.1383	0.407	
ACC-PCC	0.0546	0	–	0.0779	0.611	
PCC-STG	0.0551	0.0710	0.841	0.0709	0.019	
STG-AG	0	0	–	0	–	
AG-IPL	0.6478	0.6209	0.635	0.6226	0.075	
IPL-MTG	0.1289	0.1276	0.839	0.1199	0.699	
MTG-MFG	0.0447	0.0817	0.125	0	–	
MFG-ACC	0.6874	0.7952	0.575	0.6373	0.193	
SFG-PCC	0.1176	0.0954	0.119	0.0953	0.623	
ACC-STG	0.0823	0.0818	0.774	0.1167	0.053	
PCC-AG	0.2077	0.2081	0.831	0.2145	0.752	
STG-IPL	0.0504	0	–	0	–	
AG-MTG	0.2146	0.2176	0.856	0.2438	0.648	
IPL-MFG	0.0420	0.0632	0.742	0	–	
MTG-SFG	0.0670	0.0806	0.248	0.0631	0.617	
MFG-PCC	0	0	–	0	–	
SFG-STG	0.0477	0	–	0.0975	0.161	
ACC-AG	0.0516	0	–	0	–	
PCC-IPL	0.2443	0.2363	0.684	0.2482	0.742	
STG-MTG	0.2273	0.2622	0.396	0.2133	0.869	
AG-MFG	0	0	–	0	–	
IPL-SFG	0	0	–	0	–	
MTG-ACC	0	0	–	0	–	
MFG-STG	0.0755	0.0867	0.636	0.1219	0.195	
SFG-AG	0	0	–	0.0475	–	
ACC-IPL	0.1461	0.1228	0.395	0.1492	0.953	
PCC-MTG	0.0685	0.0504	0.938	0.0843	0.872	

The maximum of the 4 pairwise connections obtained between the two ROIs located in both hemispheres was defined as the connectivity between the two nodes (one sample *t*-test, *P*<0.05). Significant alterations induced by acupuncture at ST36 relative to NAP were based on the paired *t*-test (*P*<0.05) or with the emergence/suspension of functional connectivity following acupuncture compared with the resting state.

**Figure 5:**
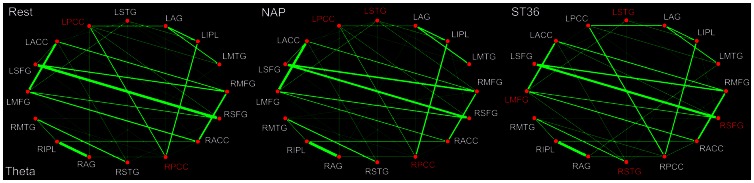
DMN hub configurations preceding acupuncture and following acupuncture at ST36 or NAP within the theta band (P<0.05, FDR corrected). The DMN hubs are indicated in red which are denoted as at least one standard deviation greater than the average degree of the network. The strength of the functional connectivity was expressed by the thickness of the green line. L, left; R, right; MFG, medial frontal gyrus; SFG, superior frontal gyrus; ACC, anterior cingulate cortex; PCC, posterior cingulate cortex/precuneus; STG, superior temporal gyrus; AG, angular gyrus; IPL, inferior parietal lobule; MTG, middle temporal gyrus.

Compared with the DMN hub configuration during the resting state in the delta band, it was recognized furthermore that the PCC constituted the only hub within the theta band (4–8 Hz). What's more, the most interesting difference lies in the fact that, following sham acupuncture at NAP, the bilateral PCC were still remained among the core hub regions, however, neither of them served as a hub following the verum intervention at ST36 (Fig. 5). Meanwhile, the STG was emerged to act as a network hub following either ST36 or NAP stimulation. In addition, it was remarkable that the SFG was specifically discerned as a DMN hub for the ST36 group. Following acupuncture at NAP, the connectivity between MTG and MFG was detected to be enhanced, while in the ST36 group, the relations between STG and AG, PCC and IPL, AG and MFG were found to be emerged, while the connectivity between AG and IPL was significantly decreased. Interestingly, the connection between IPL and SFG was detected to be strengthened following either acupuncture (Table 4).

**Table 4:** The functional connectivity between the DMN key nodes and their alterations induced by acupuncture within the theta band (4–8 Hz).

Functional Connectivity	Theta				
	Rest	NAP	*P*-value	ST36	*P*-value
MFG-SFG	0.3450	0.3848	0.405	0.3490	0.894
SFG-ACC	0.0922	0.1727	0.105	0.1204	0.142
ACC-PCC	0.0760	0.0691	0.055	0.0841	0.317
PCC-STG	0	0	–	0	–
STG-AG	0	0	–	0.0596	–
AG-IPL	0.6085	0.6289	0.064	0.2721	0.000
IPL-MTG	0.1433	0.1499	0.779	0.1399	0.833
MTG-MFG	0	0.0452	–	0	–
MFG-ACC	0.4756	0.5512	0.772	0.4608	0.132
SFG-PCC	0.0850	0.0883	0.314	0.0694	0.854
ACC-STG	0.0753	0.0841	0.714	0.0929	0.302
PCC-AG	0.2061	0.1669	0.208	0.1778	0.269
STG-IPL	0.0922	0.0818	0.545	0.0895	0.685
AG-MTG	0.2387	0.2929	0.057	0.2614	0.544
IPL-MFG	0	0	–	0	–
MTG-SFG	0.0553	0.0598	0.340	0.0679	0.533
MFG-PCC	0	0	–	0	–
SFG-STG	0.0472	0.0641	0.301	0.0614	0.463
ACC-AG	0.	0	–	0	–
PCC-IPL	0.2790	0.3263	0.091	0.5980	0.000
STG-MTG	0.2744	0.3002	0.628	0.2436	0.921
AG-MFG	0	0	–	0.0455	–
IPL-SFG	0	0.0410	–	0.0498	–
MTG-ACC	0	0	–	0	–
MFG-STG	0.0832	0.0817	0.818	0.0971	0.807
SFG-AG	0	0	–	0	–
ACC-IPL	0.1580	0.1509	0.981	0.1656	0.988
PCC-MTG	0.0773	0.0549	0.716	0.0904	0.998

The maximum of the 4 pairwise connections obtained from the two ROIs located in both hemispheres was defined as the connectivity between the two nodes (one sample *t*-test, *P*<0.05). Significant alterations induced by acupuncture at ST36 relative to NAP were based on the paired *t*-test (*P*<0.05) or with the emergence/suspension of functional connectivity following acupuncture compared with the resting state.

**Figure 6:**
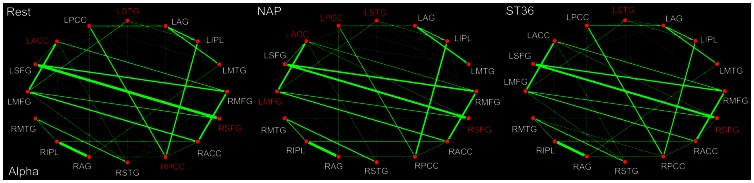
DMN hub configurations preceding acupuncture and following acupuncture at ST36 or NAP within the alpha band (P<0.05, FDR corrected). The DMN hubs are indicated in red which are denoted as at least one standard deviation greater than the average degree of the network. The strength of the functional connectivity was expressed by the thickness of the green line. L, left; R, right; MFG, medial frontal gyrus; SFG, superior frontal gyrus; ACC, anterior cingulate cortex; PCC, posterior cingulate cortex/precuneus; STG, superior temporal gyrus; AG, angular gyrus; IPL, inferior parietal lobule; MTG, middle temporal gyrus.

Among the 5 frequency bands, it seems that there are the most hubs within the alpha band (8–13 Hz) during the resting state (Fig. 6), involving the ACC, STG, PCC as well as SFG. In line with the theta band, it is notable that the left STG served robustly as network hubs following either sham or verum acupuncture. Besides, it was also illustrated that the PCC acted as a hub specifically following the sham acupuncture. Moreover, what's different between the theta and alpha band is that the SFG not only emerged as a hub for the ST36 group, but also formed a network hub for the NAP group in the alpha band. In addition, it was noted that the ACC together with MFG formed the DMN hubs following acupuncture at NAP, none of which, on the contrary, was designated for the ST36 group. Moreover, the relations between ACC and PCC, STG and AG as well as SFG and STG were weakened, while the connections between AG and MTG, MFG and PCC, MTG and ACC were found to be strengthened following the sham stimulation. In the ST36 group, the connectivity between SFG and PCC was decreased while the relations between AG and MFG, SFG and AG were increased (Table 5).

**Table 5:** The functional connectivity between the DMN key nodes and their alterations induced by acupuncture within the alpha band (8–13 Hz).

Functional Connectivity	Alpha				
	Rest	NAP	*P*-value	ST36	*P*-value
MFG-SFG	0.3511	0.3768	0.564	0.3491	0.988
SFG-ACC	0.0997	0.1573	0.066	0.1103	0.078
ACC-PCC	0.0908	0.0778	0.034	0.1063	0.403
PCC-STG	0	0	–	0	–
STG-AG	0.0303	0	–	0.0800	0.148
AG-IPL	0.6003	0.6231	0.262	0.6070	0.768
IPL-MTG	0.1620	0.1753	0.362	0.1583	0.487
MTG-MFG	0	0	–	0	–
MFG-ACC	0.4672	0.3990	0.667	0.4492	0.116
SFG-PCC	0.0489	0.0460	0.912	0	–
ACC-STG	0.0925	0.0929	0.496	0.0908	0.684
PCC-AG	0.2084	0.1649	0.111	0.1593	0.157
STG-IPL	0.1000	0.0991	0.711	0.0984	0.689
AG-MTG	0.2778	0.3513	0.007	0.2971	0.731
IPL-MFG	0	0	–	0	–
MTG-SFG	0.0649	0.0544	0.482	0.0733	0.721
MFG-PCC	0	0.0451	–	0	–
SFG-STG	0.0388	0	–	0.0580	0.299
ACC-AG	0	0	–	0	–
PCC-IPL	0.3218	0.3922	0.197	0.3020	0.606
STG-MTG	0.2581	0.2926	0.501	0.2536	0.616
AG-MFG	0	0	–	0.0524	–
IPL-SFG	0.0604	0.0544	0.697	0.0622	0.696
MTG-ACC	0	0.0556	–	0	–
MFG-STG	0.1160	0.0957	0.155	0.1151	0.889
SFG-AG	0	0	–	0.0543	–
ACC-IPL	0.1708	0.1638	0.479	0.1697	0.749
PCC-MTG	0.0588	0.0508	0.633	0.0955	0.379

The maximum of the 4 pairwise connections obtained from the two ROIs located in both hemispheres was defined as the connectivity between the two nodes (one sample *t*-test, *P*<0.05). Significant alterations induced by acupuncture at ST36 relative to NAP were based on the paired *t*-test (*P*<0.05) or with the emergence/suspension of functional connectivity following acupuncture compared with the resting state.

**Figure 7:**
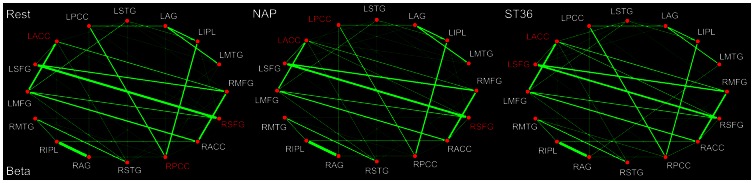
DMN hub configurations preceding acupuncture and following acupuncture at ST36 or NAP within the beta band (P<0.05, FDR corrected). The DMN hubs are indicated in red which are denoted as at least one standard deviation greater than the average degree of the network. The strength of the functional connectivity was expressed by the thickness of the green line. L, left; R, right; MFG, medial frontal gyrus; SFG, superior frontal gyrus; ACC, anterior cingulate cortex; PCC, posterior cingulate cortex/precuneus; STG, superior temporal gyrus; AG, angular gyrus; IPL, inferior parietal lobule; MTG, middle temporal gyrus.

To some extent similarity was found for the DMN hub configurations during the resting state between the beta band (13–30 Hz) and the alpha band (Fig. 7). First of all, the PCC was consistently identified as a hub. In addition, the ACC together with SFG was also designated for the beta band. Following either verum or sham acupuncture, it is noticeable that little was influenced on the role played by ACC and SFG for the DMN hub configurations. However, compared with the NAP group, the PCC did not serve as a network hub any more following acupuncture at ST36. There are also some shared alterations of the functional connectivity induced by acupuncture between the beta and alpha band. Following acupuncture at NAP, the connection between ACC and PCC, SFG and STG as well as AG and MFG were detected to be reduced, while the relations between AG and MTG, MTG and ACC, SFG and AG were enhanced. After stimulation at ST36, the connection between IPL and SFG was decreased, while the relations between STG and AG, SFG and AG were illustrated to be increased (Table 6).

**Table 6:** The functional connectivity between the DMN key nodes and their alterations induced by acupuncture within the beta band (13–30 Hz).

Functional Connectivity	Beta				
	Rest	NAP	*P*-value	ST36	*P*-value
MFG-SFG	0.3274	0.3446	0.725	0.3271	0.957
SFG-ACC	0.0915	0.1372	0.198	0.1281	0.221
ACC-PCC	0.0815	0.0717	0.023	0.0789	0.843
PCC-STG	0	0	–	0	–
STG-AG	0	0	–	0.0575	–
AG-IPL	0.6204	0.6424	0.256	0.6178	0.857
IPL-MTG	0.1534	0.1577	0.392	0.1481	0.631
MTG-MFG	0	0	–	0	–
MFG-ACC	0.4636	0.4153	0.107	0.4319	0.091
SFG-PCC	0.0642	0.0573	0.382	0.0529	0.443
ACC-STG	0.0914	0.0904	0.364	0.0897	0.598
PCC-AG	0.1979	0.1630	0.169	0.1771	0.392
STG-IPL	0.0969	0.0850	0.425	0.0878	0.441
AG-MTG	0.2357	0.3116	0.004	0.2517	0.750
IPL-MFG	0	0	–	0	–
MTG-SFG	0.0539	0.0491	0.793	0.0696	0.159
MFG-PCC	0	0	–	0	–
SFG-STG	0.0360	0	–	0.0458	0.694
ACC-AG	0	0	–	0	–
PCC-IPL	0.3118	0.3708	0.127	0.3059	0.610
STG-MTG	0.2575	0.2738	0.970	0.2535	0.667
AG-MFG	0.0429	0	–	0.0468	0.481
IPL-SFG	0.0446	0.0507	0.537	0	–
MTG-ACC	0	0.0514	–	0	–
MFG-STG	0.1121	0.0987	0.727	0.1020	0.642
SFG-AG	0	0.0458	–	0.0406	–
ACC-IPL	0.1566	0.1609	0.474	0.1405	0.595
PCC-MTG	0.0624	0.0533	0.776	0.0934	0.458

The maximum of the 4 pairwise connections obtained from the two ROIs located in both hemispheres was defined as the connectivity between the two nodes (one sample *t*-test, *P*<0.05). Significant alterations induced by acupuncture at ST36 relative to NAP were based on the paired *t*-test (*P*<0.05) or with the emergence/suspension of functional connectivity following acupuncture compared with the resting state.

**Figure 8:**
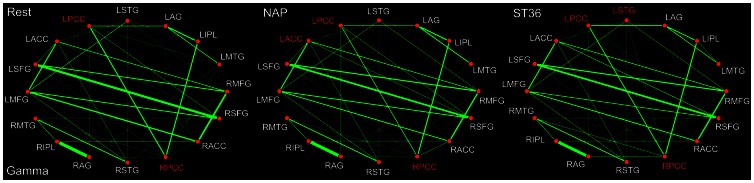
DMN hub configurations preceding acupuncture and following acupuncture at ST36 or NAP within the gamma band (P<0.05, FDR corrected). The DMN hubs are indicated in red which are denoted as at least one standard deviation greater than the average degree of the network. The strength of the functional connectivity was expressed by the thickness of the green line. L, left; R, right; MFG, medial frontal gyrus; SFG, superior frontal gyrus; ACC, anterior cingulate cortex; PCC, posterior cingulate cortex/precuneus; STG, superior temporal gyrus; AG, angular gyrus; IPL, inferior parietal lobule; MTG, middle temporal gyrus.

In line with other frequency bands, the PCC was as well shown dominantly as the default mode network hub during the resting state within the gamma band (30–48 Hz). Following acupuncture, for either verum or sham stimulation, it is of interest to find that there was little acupuncture-induced modulation over the role played by PCC in the DMN hub configurations (Fig. 8). The PCC as well as the ACC was illustrated to be the network hubs following sham acupuncture. On the contrary, apart from the PCC, the STG was denoted specifically to work as a hub following acupuncture at ST36. Following acupuncture at either NAP or ST36, only a few significant alterations of functional connectivity were demonstrated within the gamma band. For the NAP group, the connection between MTG and SFG was decreased, while the connectivity between MTG and AG was significantly increased. For the ST36 group, significant increased connectivity was detected only between PCC and STG (Table 7).

**Table 7:** The functional connectivity between the DMN key nodes and their alterations induced by acupuncture within the gamma band (30–48 Hz).

Functional Connectivity	Gamma				
	Rest	NAP	*P*-value	ST36	*P*-value
MFG-SFG	0.2814	0.2980	0.669	0.2909	0.860
SFG-ACC	0.0775	0.1195	0.584	0.1283	0.092
ACC-PCC	0.0751	0.0760	0.191	0.0698	0.789
PCC-STG	0	0	–	0.0466	–
STG-AG	0	0	–	0	–
AG-IPL	0.6653	0.6769	0.303	0.6568	0.497
IPL-MTG	0.1366	0.1321	0.551	0.1397	0.796
MTG-MFG	0	0	–	0	–
MFG-ACC	0.3825	0.3614	0.495	0.3610	0.239
SFG-PCC	0.0842	0.0868	0.672	0.0718	0.583
ACC-STG	0.1050	0.1060	0.346	0.1039	0.592
PCC-AG	0.1843	0.1504	0.120	0.1756	0.578
STG-IPL	0.0594	0.0424	0.227	0.0517	0.066
AG-MTG	0.1843	0.2440	0.009	0.1883	0.828
IPL-MFG	0	0	–	0	–
MTG-SFG	0.0448	0	–	0.0561	0.125
MFG-PCC	0	0	–	0	–
SFG-STG	0	0	–	0	–
ACC-AG	0	0	–	0	–
PCC-IPL	0.2886	0.3226	0.127	0.2845	0.942
STG-MTG	0.2578	0.2568	0.166	0.2395	0.928
AG-MFG	0	0	–	0	–
IPL-SFG	0	0	–	0	–
MTG-ACC	0	0	–	0	–
MFG-STG	0.1073	0.0953	0.226	0.0930	0.611
SFG-AG	0	0	–	0	–
ACC-IPL	0.1130	0.1176	0.772	0.0976	0.491
PCC-MTG	0.0489	0.0533	0.772	0.0841	0.265

The maximum of the 4 pairwise connections obtained from the two ROIs located in both hemispheres was defined as the connectivity between the two nodes (one sample *t*-test, *P*<0.05). Significant alterations induced by acupuncture at ST36 relative to NAP were based on the paired *t*-test (*P*<0.05) or with the emergence/suspension of functional connectivity following acupuncture compared with the resting state.

## Discussion

To the best of our knowledge, it is the first study to explore spatiotemporally the acupuncture effects on the DMN hub configurations within conventional frequency bands (delta, theta, alpha, beta and gamma) following stimulation at ST36, using a nearby non-acupoint (NAP) as a control, by combining fMRI and MEG analysis.Results exhibits differential alterations within the hub configurations of the DMN during the post-stimuli acupuncture resting state.

### DMN hub configurations during the resting state

Among all DMN core regions, the PCC has been extensively denoted as the pivotal role in connecting other DMN brain regions for transmitting information under the mentioned cognitive processes, or optimizing the connectivity pattern to reduce cost of wiring and resources [23], [29]–[30]. In the present study, by combining fMRI and MEG techniques, we not only confirmed the crucial function of the PCC in the DMN, but further extend previous knowledge about its hub configuration in the frequency domains. It is worthy to mention that the PCC constitutes the most attention-grabbing region as it is the only one which acted as a DMN hub consistently across the 5 conventional frequency bands (delta, theta, alpha, beta and gamma rhythm). Meanwhile, in line with previous fMRI studies, the IPL [79]–[80] and ACC, SFG [28] were validated as DMN hubs during the resting state. Moreover, with the advantage of MEG in providing amount of details in temporal dimension, it was further revealed that their crucial functions were exerted not across the 5 bands but within specific rhythms, especially with delta band for IPL and alpha, beta bands for both ACC and SFG.

### Differential hub configurations following verum and sham acupuncture

Recently a number of noninvasive sham controls have been developed and tested [38], [81]–[82]. While these hold promise in some respects, they also have limitations in what they can be used for, thus they should be used only when it is clear that their use matches the question for which sham treatment model is being selected [83]. Sham acupuncture is proved to a reasonable placebo control in many acupuncture fMRI setting, and can effectively reduce the subjects' bias toward the stimulation. In the current study, we also employed sham acupuncture as a control model. The comparison of the verum acupuncture, compared with the sham control, was expected to reveal the acupoint-specific response in the human brain and the observed differences between these two conditions may constitute a specific physiological effect.

As illustrated in aforementioned results (Fig. 4–8), the PCC constitute one of the most noteworthy DMN hubs with saliently differential alterations following either verum or sham acupuncture. Following sham acupuncture at NAP, the PCC remained to serve consistently as DMN hub across all 5 frequency bands. However, it is interesting to find out that the PCC was to a large extent regulated in specific bands by the verum acupuncture at ST36. Actually, it is only within delta and gamma bands that the PCC still acted as a DMN hub. As implicated typically in previous neuroimaging studies, the PCC served as not only a DMN hub [28], but a core region for the whole brain network when engaging more brain regions within the whole brain network [23]. Recently, it is speculated that the brain regions are organized into interleaved networks to accomplish various functions or healing effects when a neural intervention is triggered [7]. Since DMN involves a mode of preparedness and alertness of possible changes in the internal milieu as well as external environment, the integrity of connectivity between the DMN and other brain networks may be central to the balance of brain functions [84]. Previous studies have shown alterations in the generalized activity and functional connectivity of the DMN in patients with various kinds of disorders [85]–[87]. Following stimulation at pain-control acupoints, it is demonstrated as well that there is increased DMN connectivity with pain related regions [40]. This suggests that specific brain networks may facilitate a correspondence between acupuncture stimulation and the central nervous system. Moreover, it has been proven, following acupuncture at vision-related acupoint, that the PCC performed intensive connections with other discrete core regions in vision networks [7]. Taken into account the ST36 is a commonly used acupoint for pain control in clinical practice, it is postulated that PCC would engage extensive interactions with the neural networks for both pain transmission and perception [88]–[89]. The connectivity was therefore believed to be enhanced between the PCC and those core regions within the pain matrix [90]. As a result, the role as a DMN hub played consistently by the PCC across the 5 frequency bands may be to some extent inhibited, supposed to be reallocated in order to balance the energy for the whole brain network to better exert the analgesia effect. In addition, it is for the first time revealed that the physiological regulations induced by acupuncture were dependent on the specific rhythms of neural activity. Compared with sham acupuncture, the acupuncture-induced modulation for PCC was mainly confined within the theta, alpha and beta bands.

Another striking finding is that, regarding to other core regions, to a certain extent shared DMN hub configuration patterns were observed following acupuncture at either ST36 or NAP. Given that acupuncture is a complex intervention that is intimately intertwined with placebo, patients, and practitioners. Therefore, it is logical that acupuncture may induce both specific and non-specific effects which contribute to its therapeutic effects [6]. That may support the clinical facts that acupuncture at sham points can also provide partial analgesia in chronic pain [91]. Moreover, in our present study, while the locations of these connectivity changes had overlap between verum and sham there were apparent differences as to the specific frequency bands between post-acupuncture modulation and post-sham modulation. Although STG were not among the DMN core regions during the resting state, it began to serve as a hub following both kinds of acupuncture. Nevertheless, it is implicated that sham intervention exerts such effect within only theta and alpha bands, while verum acupuncture produces additional influence for the gamma band. Likewise, the MFG demonstrates as well the acupuncture-evoked impacts on hub configurations within theta band following the verum intervention, in relative to the alpha band for the control group. In addition, the SFG constitutes as a DMN hub within the alpha and beta bands during the resting state. As illustrated in our results, the sham acupuncture seems to exert no effect on its hub configuration, remaining as a hub within the alpha and beta bands. Following verum intervention, however, the SFG served as a hub within not only these two bands, but additionally delta and theta rhythms.

In conclusion, the present study demonstrated differential alterations within 5 conventional frequency bands (delta, theta, alpha, beta and gamma) of DMN hub configurations following acupuncture at ST36 relative to NAP. With the complementary advantage of fMRI and MEG, we are able to explore spatiotemporally the specific biological mechanism underlying acupuncture, especially on the modulation of PCC as a DMN hub within the theta, alpha and beta bands. Overall, though it is a preliminary work, our results may provide additional evidence for the specificity of acupoint. We hope that this article serves as an introduction that will help to explore this fascinating topic in more depth and, as a consequence, shed more light on the specific neurophysiological mechanisms of acupuncture.
